# The Human Brain Online: An Open Resource for Advancing Brain Research

**DOI:** 10.1371/journal.pbio.1001453

**Published:** 2012-12-27

**Authors:** Sara Ball, Terri L. Gilbert, Caroline C. Overly

**Affiliations:** Allen Institute for Brain Science, Seattle, Washington, United States of America

## Abstract

This community page describes the database and associated Web application that comprise the Allen Human Brain Atlas, an open online resource that integrates genomic and anatomic human brain data.

## Introduction

With an estimated 86 billion neurons [1] and about a trillion synapses per cubic centimeter of cortex [2], the human brain is arguably the most complex system in the human body, and it is the seat of diseases and disorders that affect an estimated one billion people worldwide [3]. Yet the human brain remains poorly understood. Model systems are essential to progress in neuroscience, but a true understanding of the human brain and the diseases and disorders that affect it ultimately requires analyses of the human brain itself. Human brain tissue is a rare commodity and therefore inadequately explored. Published studies point to the scarcity of high-quality postmortem human brain tissue, particularly disease-free control brains [4]; the largest brain bank in the United States reported last year that only 40–50 control brains become available each year [5]. Further hindrance lies in the fragmented nature of data from studies with human brain tissue. Brain banks typically subdivide the brain into small blocks to distribute among a variety of researchers, thus precluding holistic analyses, and data derived from such studies are focused on diverse and often nonparallel hypotheses and experimental approaches.

Here we describe an open online resource, the Allen Human Brain Atlas, which puts comprehensive, standardized data from multiple entire human brains into the hands of the global research community, along with tools for mining and making sense of that data. This resource opens new avenues for advancing research programs across disciplines that share an interest in the human brain—from neuroscience research programs based on functional MRI (fMRI) or neuropharmacology, for example, to comparative evolutionary studies and human genetics. The Allen Human Brain Atlas is a multimodal atlas of gene expression and anatomy comprising a comprehensive “all genes, all structures” array-based dataset of gene expression and complementary in situ hybridization (ISH) studies targeting selected genes in specific brain regions. All data are publicly available online (www.brain-map.org) along with a suite of integrated data visualization and mining tools that enable scientists to uncover connections between structure, function, and the brain's underlying biochemistry.

In developing the earlier Allen Mouse Brain Atlas, a genome-wide, high-resolution atlas of gene expression throughout the adult mouse brain [6], the Allen Institute for Brain Science created the infrastructure to handle high-throughput ISH, microscopy, and data processing. This expertise enabled the Allen Institute to tackle high-throughput processing of human tissue and to systematically create an atlas of spatially mapped gene expression in the human brain. In addition to decisions concerning level of resolution and project scope [7], a major challenge was to define processes for systematic dismantling and sequential partitioning of the brain to enable gathering multiple types of data from a single brain and allow reassembly of those data into a unified 3-D framework. From initial tissue procurement and processing at the front end to data integration at the other end, a number of new methods were developed to deal specifically with human tissue in this high-throughput setting. After tissue procurement—which involves obtaining consent, tissue dissection, MRI, and diffusion tensor (DTI) imaging, slabbing, and freezing of the tissue all within a very short window of time—rigorous steps are taken for sample inclusion, such as assessment of tissue/RNA quality, gross and microneuropathology, toxicology, and medical history research. New workflows allowed for sampling of specific anatomic regions for microarray analysis and mapping those locations back into the 3-D brain space determined by the MRI. Detailed descriptions of scientific and informatics methods are available in the whitepapers under the Documentation tab of the online atlas.

## Data and Tools

**Figure 1:**
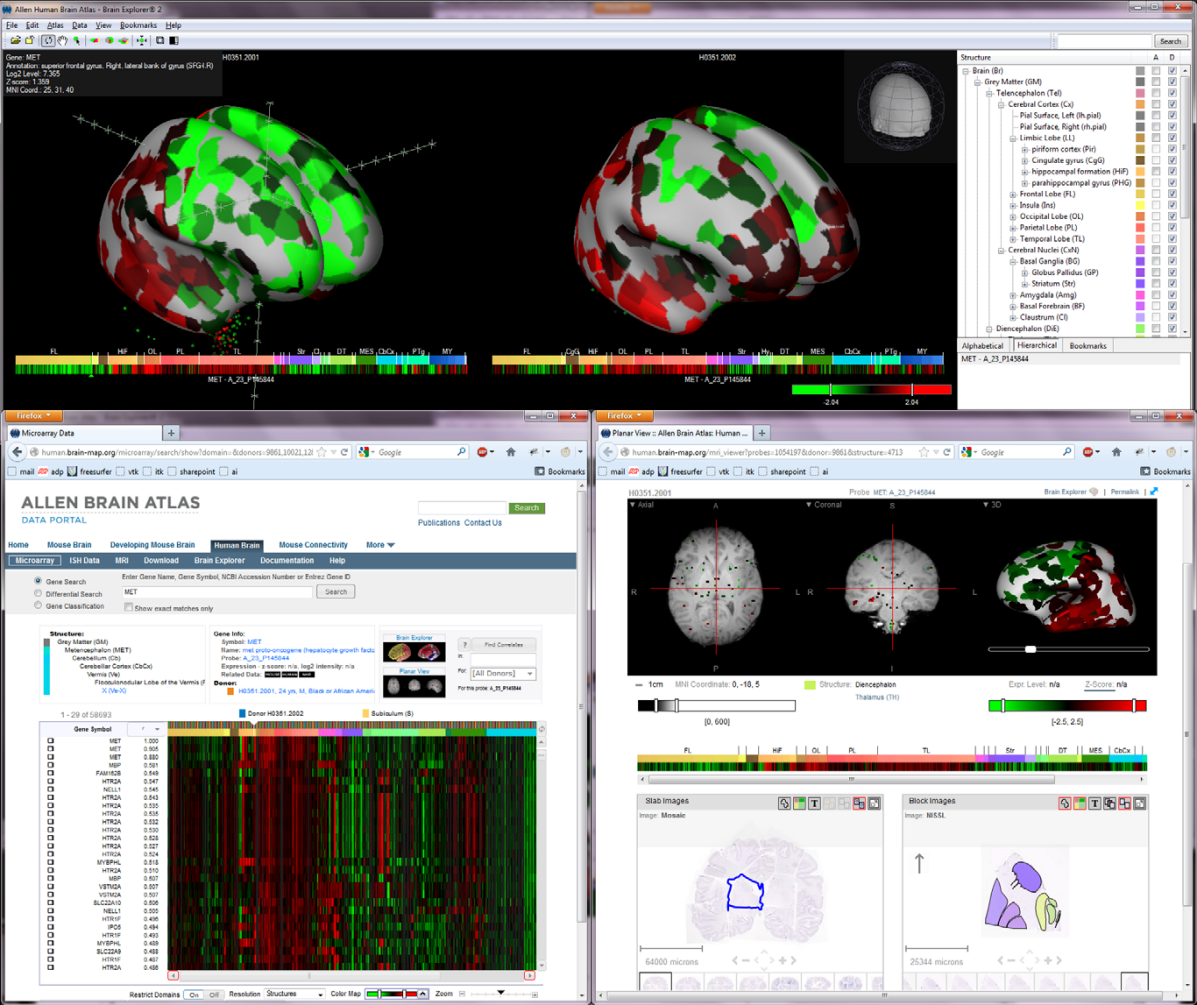
The Allen Human Brain Atlas contains multimodal data integrated into a unified 3-D framework with search and visualization features allowing one to journey through the brain readily climbing up and down levels of resolution. See it in action: Check out video tutorials on the Allen Human Brain Atlas and Brain Explorer® 3-D Viewer. Key Features: An “all genes, all structures” gene expression survey in multiple adult control brains. • >62,000 gene probes per profile. • ∼500 samples per hemisphere across cerebrum, cerebellum, and brainstem. • Data mapped with histology into unified 3-D anatomic framework based on MRI. High-resolution ISH image data covering selected genes in specific brain regions. • Subcortex Study: 55 genes across subcortical regions and 10 additional genes in hypothalamus in one male and one female donor. • Cortex Study: 1,000 genes in visual and temporal cortices in multiple adult control brains. • Schizophrenia Study: 60 genes in dorsolateral prefrontal cortex of over 50 control and schizophrenia cases. • Autism Study: 25 genes in frontal, temporal and occipital cortical regions of 11 control and 11 autism cases. • Neurotransmitter Study: Selected neurotransmitter system genes in major cortical and subcortical areas in adult control brains. MRI data for brains used for all microarray and some ISH analyses. Search and viewing tools, including: • Brain Explorer® 3-D viewer. • Heatmap viewer for exploring microarray data across genes and brain regions. • Gene-based and anatomic search features. • Multiplanar MRI viewer with gene expression overlay. • Linked viewing of MRI, gene expression, histology, anatomic delineations.

The Allen Human Brain Atlas includes genome-wide microarray data for approximately 500 discrete anatomic regions per hemisphere of the adult human brain, along with ISH data covering select genes in specific brain regions and complementary anatomic data. With more than 100 million microarray expression data points from three brains and over 46,000 ISH images to date, the depth and breadth of data transcend the resources available to traditional laboratories, allowing scientists to easily explore beyond the streetlamp and into the shadows. The entire dataset—including MRI, DTI, histology, immunohistochemistry, ISH, transcriptome data from microarray, and anatomic annotation—is viewable online and downloadable for offline use and analysis. The associated Web application includes unique search and visualization tools providing multiple entry points into the data, accompanied by an interactive 3-D viewer that allows you to spin, slice, and search each entire brain in the virtual world (Figure 1). With more brains in the pipeline and a large ISH study of neurotransmitter system genes underway, new data will be added to the Atlas into 2013. Initially launched online in May 2010, the Allen Human Brain Atlas is already a widely used resource with approximately 7,000 unique visitors each month worldwide.

The Allen Human Brain Atlas database and associated Web application were designed as a bridging resource, with multiple data types offering multiple entry points for researchers coming from different areas of expertise with different questions. Whether accessing the Allen Human Brain Atlas from a gene-centric perspective or a structural or functional point of view, the variety of data—histology, annotation, genomics, and MRI—provides a launchpad for discovery.

## Atlas in Action

Data from the Allen Human Brain Atlas have revealed that 84% of all genes in the human genome are expressed somewhere in the brain [8], and the Atlas catalogs each of these genes with a quantitative fingerprint mapping their expression location(s). Among its many uses, consider genome-wide association studies (GWAS) and other human genetics studies churning out growing lists of candidate genes for diseases or other traits (e.g., as of June 2012, 91 genes and 7 intergenic regions have been associated with schizophrenia [9]); the Atlas offers a readily available resource to help sort and prioritize these lists and understand more about the biology of what the genes are doing in the brain. Two recent studies used data in the Allen Human Brain Atlas to examine genes implicated in Alzheimer's disease and autism spectrum disorders by modeling gene interactions and analyzing gene networks, respectively [10],[11].

As another example, from an anatomic perspective, fMRI studies reveal activation areas associated with particular behaviors, cognitive processes, diseases, or genetic profiles, highlighting interest in a certain region of the brain. Detailed gene expression information for that region provides a path to a more complete understanding of its underlying biochemistry, potentially revealing what distinguishes it from other brain areas and helping to elucidate the biological processes that relate to the phenotype of interest. Further, a recent review points to the potential of fMRI studies to speed the drug discovery process for central nervous system diseases, particularly via use of brain imaging biomarkers [12]. The Atlas can take this proposed process a step further by revealing associations between regions or imaging biomarkers of interest and genes at work in those areas.

As most work in neuroscience is conducted in model systems, the Atlas also provides a platform to help verify and translate such work into a human context. A recent paper scratches the surface of the types of comparisons that can be made between the mouse and human brain using publicly available online data. Among other observations, the study points to a 79% similarity in expression of approximately 1,000 genes in the visual cortex of the mouse and human brain, as well as identifying distinct molecular markers specific to each species [13].

The discovery of global patterns and general principles within the brain is another critical step toward understanding how it works. A study published last year suggests that spatial gene expression data are integral in informing gene–phenotype association predictions [14]. The experimental design in this study was first tested as a proof-of-concept study with Allen Mouse Brain Atlas [15], then repeated with the Allen Human Brain Atlas, whereby gene expression data were used to predict promising candidate genes for genetic susceptibility to seizures. Furthermore, researchers have used the Allen Mouse Brain Atlas to find that brain regions with similar patterns of gene expression have similar connectivity profiles [16]; this type of work can now be done directly in the human brain.

## Beyond the Atlas

The increasing amount of data and tools available through the Allen Brain Atlas portal are only as valuable as the applications of the scientists who use them, so ensuring their usefulness is a priority of the Allen Institute. To this end, the Allen Institute offers both Web-based and in-person training sessions, as well as video tutorials, to help researchers become more adept at using these resources. Additionally, the Allen Human Brain Atlas has been designed to facilitate cross-referencing with other Allen Brain Atlas resources for comparative studies among species and across development. A growing hub for extensive, systematically generated datasets and sophisticated data mining and visualization tools, the Allen Brain Atlas portal provides public access to a collection of resources for exploring the central nervous system. These include gene expression atlases of the adult and developing mouse brain, mouse spinal cord, adult and developing human brain and the rhesus macaque brain, as well as a recently launched mouse brain connectivity atlas. The entire suite of Allen Brain Atlas resources, with embedded crosslinks to related data and global search across all datasets, is available at http://www.brain-map.org.

Furthermore, there is a growing number of other online resources that can be used synergistically with the Allen Human Brain Atlas to address questions about human brain function, organization, and disease, and examples of combined use are just beginning to emerge. Studies have used the Atlas with protein–protein interaction data from the Human Protein Reference Database (HRPD) [17] and with the Gene Expression Omnibus (GEO) [18] to investigate gene interactions associated with Alzheimer's disease [10] and the genetic origins of seizure susceptibility [14], respectively. Another recent study used the Autism Genetic Resource Exchange (AGRE) [19] SNP data followed by the Allen Human Brain Atlas to help identify and localize key genes for predictive diagnosis of autism spectrum disorders (ASDs) [20]. Other complementary resources include imaging databases such as the Human Connectome Project and the Alzheimer's Disease Neuroimaging Initiative (ADNI) database, as well as numerous molecular, anatomy, human genetics, and disease-specific resources [21]. The release of the full Allen Brain Atlas application programming interface API in June 2012 opened the door for more extensive neuroinformatics analyses and integration with such resources by the end user community.

## Abbreviations

DTI: diffusion tensor imaging
GWAS: genome-wide association studies
fMRI: functional magnetic resonance imaging
ISH: in situ hybridization
MRI: magnetic resonance imaging
